# Presence of toxin-producing bacterial pathogens and associated risk factors in neonatal diarrhea of piglets on commercial sow farms in Vietnam

**DOI:** 10.14202/vetworld.2025.1288-1296

**Published:** 2025-05-21

**Authors:** Duy Tien Do, Tram Thi Ngoc Ngo, Huong Dieu Vu, Nhat Minh Duong, Sittikorn Traiyarach, Danh Cong Lai, Joaquin Miguel Escuder

**Affiliations:** 1 Department of Infectious Diseases and Veterinary Public Health, Faculty of Animal Science and Veterinary Medicine, Nong Lam University, HCMC, Thu Duc City, Ho Chi Minh City, Vietnam; 2 The Animal Biomedical Research Laboratories, Nong Lam University, HCMC, Vietnam; 3 HIPRA Vietnam, Ho Chi Minh City, Vietnam; 4 HIPRA, Amer (Girona), Spain; 5 Nebraska Center for Virology, University of Nebraska-Lincoln, Lincoln, USA

**Keywords:** bacterial toxins, *Clostridioides difficile*, *Clostridium perfringens*, *Escherichia coli*, farm management, multiplex quantitative polymerase chain reaction, neonatal piglet diarrhea, Vietnam, risk factors

## Abstract

**Background and Aim:**

Neonatal piglet diarrhea (NPD) remains a significant challenge in the swine industry, contributing to elevated pre-weaning mortality, reduced productivity, and increased economic losses. In Vietnam, despite the growing importance of commercial pig production, comprehensive studies investigating the epidemiology of NPD and associated bacterial pathogens are lacking. This study aimed to assess the prevalence of *Escherichia coli*, *Clostridium perfringens*, and *Clostridioides difficile* and to identify risk factors contributing to NPD on Vietnamese sow farms.

**Materials and Methods:**

A cross-sectional study was conducted on 40 commercial sow farms across North, Central, and South Vietnam between August and December 2023. Structured questionnaires captured data on farm characteristics, management practices, and health interventions. Fecal samples from symptomatic piglets aged 1–14 days were pooled and analyzed using multiplex quantitative polymerase chain reaction to detect virulence genes of *E. coli* (F4, F5, F6, LT), *C. perfringens* (alpha, beta, and epsilon toxins), and *C. difficile* (toxins A and B). Logistic and ordinal regression models were applied to assess associations between risk factors and pathogen prevalence.

**Results:**

All farms tested positive for at least one pathogen. *C. perfringens* was the most prevalent (97.37%), followed by *E. coli* (46.49%) and *C. difficile* (39.47%). Co-infections involving multiple pathogens were common (64.91%), with *C. perfringens* consistently present in all mixed infections. Key virulence genes detected included LT (35.96%), alpha toxin (95.61%), and toxin A (20.17%). Significant risk factors included farm type, region, weaning performance, and peripartum antibiotic administration route. Notably, farms using mixed-feed antibiotics exhibited higher *E. coli* prevalence. Larger farms and those practicing early piglet relocation also showed increased pathogen diversity.

**Conclusion:**

This study presents the first national-scale assessment of bacterial pathogens in NPD across Vietnamese sow farms. The findings highlight the high burden of toxin-producing bacteria, frequent co-infections, and multiple farm-level risk factors. Interventions such as targeted vaccination, optimized antibiotic use, improved weaning practices, and enhanced regional surveillance are essential for mitigating NPD impacts and improving piglet health outcomes in Vietnam.

## INTRODUCTION

Neonatal piglet diarrhea (NPD) is a significant concern in the swine industry and represents one of the most common and economically detrimental diseases affecting piglets [1]. NPD leads to considerable production losses, including elevated pre-weaning mortality, reduced growth performance, and increased costs related to treatment. Although Johansen *et al*. [2], Kongsted *et al*. [3], and Svendsen *et al*. [4] have reported pre-weaning mortality rates due to diarrhea ranging from 5% to 24%, the financial impact on pig farms remains substantial. In Southeast Asia, particularly in Vietnam, NPD persists as a costly challenge, exacerbated by intensive farming practices, extensive antimicrobial usage, and inconsistent biosecurity measures. Recent data from Thailand and the Philippines suggest a rising incidence of NPD, alongside an increasing prevalence of multi-pathogen infections, which are often worsened by suboptimal management practices [5, 6]. In Vietnam, several bacterial pathogens have been more frequently detected in diarrheic piglets, especially on farms with deficient management systems [7]. As the Vietnamese pig industry continues to expand, controlling NPD and mitigating its associated economic burden are critical to maintaining herd health and productivity.

The etiology of NPD is multifactorial, involv-ing both infectious and non-infectious contri- butors [8, 9]. Non-infectious elements such as sow health, environmental conditions, hygiene protocols, and the piglets’ ability to consume colostrum play crucial roles in the development or exacerbation of NPD [10–12]. On the infectious side, several pathogens – including *Escherichia coli*, *Clostridium perfringens* (Types A and C), *Clostridioides difficile*, *Cystoisospora suis*, and viruses such as Porcine Epidemic Diarrhea virus – have been identified as primary agents [9, 13–15]. Importantly, field cases often involve co-infections, wherein multiple pathogens interact synergistically, resulting in more severe clinical outcomes [14].

The contribution of bacterial pathogens to NPD is increasingly recognized. *E. coli*, particularly enterotoxigenic (ETEC) strains, is a major causative agent [13, 16]. ETEC strains are defined by adhesion fimbriae that facilitate intestinal colonization and enterotoxin secretion, which induces diarrhea. Likewise, *C. perfringens* and *C. difficile* are significant contributors to neonatal diarrhea. *C. perfringens* produces a range of toxins, notably alpha (α) and beta (β), leading to severe enteritis. *C. difficile*, which produces enterotoxins TcdA and TcdB, has zoonotic potential and is associated with severe enteric disease in piglets [17, 18].

Despite the recognized impact of NPD on swine health and farm productivity, limited epidemiological data exist regarding the prevalence and risk factors of bacterial pathogens associated with NPD in Vietnam. While previous studies from other Southeast Asian countries have identified *Escherichia coli*, *C. perfringen*s, and *C. difficil*e as principal etiological agents, there remains a lack of comprehensive, region-specific investigations within Vietnamese commercial pig farming systems. Moreover, existing literature inadequately addresses the concurrent circulation of multiple pathogens and the influence of farm-level management practices, geographic variation, and antimicrobial usage on disease dynamics. This absence of integrated pathogen and risk factor profiling restricts the development of effective, evidence-based intervention strategies for the Vietnamese swine industry.

This study aims to estimate the prevalence and distribution of *E. coli*, *C. perfringens*, and *C. difficil*e in neonatal piglets exhibiting diarrheal symptoms across commercial sow farms in Vietnam. Furthermore, it seeks to identify key farm-level risk factors – including production system type, antibiotic administration routes, and geographic location – associated with pathogen occurrence and co-infection patterns. By combining molecular diagnostics with a structured farm management survey, the research intends to provide actionable insights for improving health management practices, enhancing early disease detection, and informing policy frameworks to reduce the burden of NPD in Vietnam’s swine sector.

## MATERIALS AND METHODS

### Ethical approval and Informed consent

The study was conducted in strict compliance with ethical standards approved by the Ministry of Agriculture and Rural Development of Vietnam (Protocol TCVN 8402:2010), ensuring humane and low-stress handling of all piglets involved. The farm owners and managers provided verbal consent to use the farm information for the study.

### Study period and location

The study was conducted from August to December 2023 in Vietnam, encompassing 40 commercial sow farms of varying capacities. These farms were located across three major geographic regions – North, Central, and South Vietnam – constituting a representative sample for evaluating current NPD issues in the country. All samples were analyzed at HIPRA Diagnosis (Vietnam).

### Farm information

The farms were selected based on convenience sampling methods and voluntary participation, rather than random selection criteria. A list of commercial pig farms experiencing recurrent NPD was compiled through regional veterinary networks. Farms were invited to participate based on operational scale, geographic location, and accessibility for sampling. Large farms in Vietnam often hesitate to engage in epidemiological sampling due to concerns over disease control measures. Nevertheless, we successfully accessed 40 sow farms, collectively housing approximately 138,000 sows – representing around 5% of the total Vietnamese sow population.

To ensure alignment with the study objectives, farms were required to meet inclusion criteria, including classification as a commercial farm and a documented history of recurrent NPD. All 40 farms satisfied these criteria and were enrolled in the study. In addition, none of the participating farms had implemented vaccination programs targeting *E. coli*, *C. perfringens*, or *C. difficile*. Farm owners and managers voluntarily contributed data through a structured survey questionnaire, offering valuable insights into production systems, disease prevalence, and management challenges within Vietnam’s commercial swine industry.

### Questionnaire survey

A structured questionnaire was initially pilot-tested on a subset of farms to ensure the clarity, reliability, and validity of responses regarding management practices and farm characteristics. The final version was designed to collect information on management routines and potential risk factors at two levels. The first section focused on general farm characteristics and productivity parameters (10 questions), including farm type, size, geographic region, porcine reproductive and respiratory syndrome (PRRS) status, NPD concern level, and productivity indicators such as piglets born alive, weaners per sow per year, piglet mortality rate, and average weaning weight.

The second section assessed 15 management-related variables, covering disinfection protocols, vaccination strategies for neonatal diarrhea, the application of gut feedback in sows, peripartum antibiotic use, morbidity of piglet diarrhea, incidence of mastitis and metritis, NPD frequency in younger parities, split suckling, time between farrowing and fostering, percentage of piglets moved after two days, and use of iron and coccidiostats, among others.

Farm owners and managers completed the questionnaire independently. Discrepancies were addressed through follow-up discussions to ensure consistent and accurate reporting. If both respondents provided matching answers, those responses were recorded. In cases of disagreement, the participants were asked to discuss and reach a consensus before finalizing their answers. The questionnaire included a combination of brief open-ended, multiple-choice, dichotomous, and yes/no questions. Responses related to productivity were based on average data from the preceding year. All questionnaire items and responses are detailed in Tables S1 and S2 (Supplementary data).

### Sample collection

Three litters with the most prominent NPD symptoms were selected from each farm for sample collection. Clinical signs guiding selection included watery or pasty diarrhea – typically yellow or grayish-white – commonly staining the perianal area and tail. Piglets frequently exhibited signs of dehydration, such as sunken eyes, dry skin, decreased skin elasticity, and dull or rough hair coats. To ensure a comprehensive assessment of NPD prevalence and severity on commercial farms, only litters demonstrating severe and characteristic symptoms were sampled. From each affected litter (aged 1–14 days), fecal samples were pooled from three symptomatic piglets to optimize pathogen detection efficiency and representativeness while minimizing analytical costs.

Fecal material was collected directly from the rectum using sterile swabs inserted into each symptomatic piglet. On collection, samples were preserved using FTA^®^ ELUTE cards (Whatman Inc., Florham Park, NJ, USA) to prevent DNA degradation. The cards were then refrigerated at 2°C–8°C and transported to the laboratory within 24 h. On arrival, samples were either processed immediately or stored at −20°C to preserve microbial DNA for later analysis.

### Multiplex quantitative polymerase chain reaction (qPCR)

DNA extraction was performed using an automated QIAcube system and QIAamp DNA Mini Kit (Qiacube 2.0, Qiagen, Netherlands), following the manufacturer’s protocol to ensure consistency in DNA quality and yield across all samples. Extracted DNA was immediately used for further analysis.

A validated multiplex qPCR assay (SYBR^®^ Green qPCR Kit, Qiagen, Netherlands), optimized for simultaneous detection, was employed to identify virulence genes associated with *E. coli* (F4, F5, F6, and LT toxin), *C. perfringens* (alpha, beta, and epsilon toxins), and *C. difficile* (toxins A and B) [19–21]. The selection of these pathogen-specific genes was based on their established association with NPD, as reported in prior epidemiological studies by Albini *et al*. [19], West *et al*. [22], and Houser *et al*. [23]. These virulence genes are commonly detected in pathogens responsible for gastrointestinal infections in piglets, particularly during the early postnatal period when they are most susceptible.

Samples with a cycle threshold (Ct) value greater than 38.5 were classified as negative, according to standard diagnostic thresholds [24]. Positive samples were further categorized based on Ct values, reflecting the quantity of genetic material. For *E. coli* and *C. difficile*, a Ct value above 30 was indicative of a low genetic load, while values below 30 represented a high load. For *C. perfringens* alpha toxin, a Ct threshold of 26 was applied – values exceeding 26 were interpreted as low bacterial load, whereas values below 26 indicated high genetic concentration.

### Statistical analysis

All data were analyzed using R programming software. Statistical significance was defined at p ≤ 0.05, with results showing p-values between 0.05 and 0.10 considered as indicative trends. Logistic regression analysis was employed to evaluate associations between explanatory variables and the presence of *C. perfringens* type A. In addition, ordinal regression analysis – adjusted for confounding factors such as farm type and geographic region – was conducted to examine the relationship between farm management practices and pathogen prevalence. Non-parametric tests, including the Mann–Whitney–Wilcoxon test (for binary variables) and the Kruskal–Wallis test (for variables with more than two categories), were utilized due to the non-normal distribution of prevalence data, ensuring statistical rigor and validity of inference.

## RESULTS

### Data characteristics

Survey data were collected from 40 pig farms located across the northern, central, and southern regions of Vietnam (Table S1 and S2, Supplementary data). Among these farms, 82.5% operated as closed-house systems, while 17.5% functioned as open-house systems. Seventy percentages of the surveyed farms operated as farrow-to-finish systems, with the remaining 30% raising sows and piglets up to the weaning stage. Farm sizes were clearly categorized into two groups (≤1000 sows, >1000 sows), allowing for robust comparative analysis of pathogen prevalence in relation to operational scale. Specifically, 57.5% of farms housed 1,000 sows or fewer, while 42.5% had more than 1,000 sows. The PRRS status of farms was also documented, due to its known influence on herd immunity and disease susceptibility. Among the farms, 80% were classified as PRRS-positive stable, 15% as unstable positive, 2.5% as negative, and the remaining farms had unknown PRRS status.

### Percentage of pathogens and their toxins

All farms and samples included in the study tested positive for at least one of the pathogens associated with neonatal diarrhea (Table 1). Of the 114 fecal samples analyzed, 40 (35.09%) were positive for a single pathogen, while 74 (64.91%) harbored multiple pathogens, involving either two or three agents. *C. perfringens* was detected in the highest proportion of samples (111/114, 97.37%) and was present on all 40 farms (100%). *E. coli* was found in 46.49% of samples and was present on 67.5% of the farms, while *C. difficile* was identified in 39.47% of samples and occurred on 60% of farms. The differences in pathogen detection between farms and samples were statistically significant (p < 0.001).

**Table 1:** Percentage of detected pathogens at farm and sample levels.

Positivity of pathogens	Farms (N = 40)		Samples (N = 114)	
	
	n/N	%	n/N	%
*C. perfringens*	3/40	7.5	37/114	32.46
*E. coli*	0/40	0.0	2/114	1.75
*C. difficile*	0/40	0.0	1/114	0.88
*C. perfringens + C. difficile*	10/40	25.0	23/114	20.18
*C. perfringens + E. coli*	13/40	32.5	30/114	26.32
*C. perfringens + E. coli + C. difficile*	14/40	35.0	21/114	18.42

*E. coli=Escherichia coli, C. perfringens=Clostridium perfringens, C. difficile=Clostridioides difficile*, n: number of postive farms or samples, N: total number of farms or samples surveyed

Concurrent detection of two or more pathogens was frequently observed, with *C. perfringens* being the most common in co-infected samples. The co-presence of all three pathogens was identified in 14/40 farms (35%), whereas *E. coli* and *C. difficile* were never found alone and only appeared in combination with *C. perfringens* at the farm level (Table 1).

With respect to virulence factors, this study uniquely identifies LT-toxin genes in *E. coli*, the alpha-toxin in *C. perfringens*, and the A-toxin in *C. difficile* as significantly more prevalent among diarrheal piglets than previously reported, with detection rates of 35.96%, 95.61%, and 20.17%, respectively. Adhesion factor genes (F4, F5, and F6) of *E. coli*, the beta and epsilon toxins of *C. perfringens*, as well as the B-toxin of *C. difficile*, were detected at lower frequencies (Table 2). Notably, the coexistence of two toxins within a single pathogen was common, such as LT toxin + F4 in *E. coli*, and A-toxin + B-toxin in *C. difficile*.

**Table 2:** The presence of toxins from pathogens causes neonatal diarrhea.

Pathogens	Toxins	Farms (N = 40)		Samples (N = 114)	
		n/N	%	n/N	%
*E. coli*	LT toxin	17/40	42.5	41/114	35.96
	F4	3/40	7.5	3/114	2.63
	LT toxins + F4	5/40	12.5	7/114	6.14
	LT toxins + F5	1/40	2.5	1/114	0.88
	LT toxins + F6	1/40	2.5	1/114	0.88
*C. perfringens*	Alpha	38/40	95	109/114	95.61
	Alpha + Beta	1/40	2.5	1/114	0.88
	Alpha + Epsilon	1/40	2.5	1/114	0.88
*C. difficile*	A toxin	11/40	27.5	23/114	20.17
	B toxin	2/40	5	6/114	5.26
	A + B toxins	11/40	27.5	16/114	14.03

*E. coli=Escherichia coli, C. perfringens=Clostridium perfringens, C. difficile=Clostridioides difficile*, n: number of postive farms or samples, N: total number of farms or samples surveyed

In terms of bacterial load, samples with lower Ct-values indicated higher bacterial concentrations. *C. perfringens* type A (Ct-value <26) was found on 26/40 farms (65%) and in 56/114 samples (49.12%). *C. difficile* (Ct-value <30) was detected on 10/40 farms (25%) and in 16/114 samples (14.04%), while *E. coli* (Ct-value <30) was identified on 6/40 farms (15%) and in 12/114 samples (10.53%).

### Potential risk factors for neonatal diarrhea in piglets

#### Individual pathogens and relationship with farm variables

The logistic regression model assessing risk factors and the presence of multiple pathogens revealed statistically significant associations with the following variables: “farm type,” “geographical region,” “peripartum antibiotic administration in sows,” and “average number of pigs weaned by sows” (Table 3). A novel finding was that farrow-to-finish farms exhibited a significantly higher prevalence of *C. perfringens* than those categorized as “farrowing sow” operations (p = 0.05), although no significant differences were observed for *E. coli* or *C. difficile*. Conversely, farms in the southern region had a significantly lower prevalence of *C. difficile* (p < 0.01) compared to farms in the northern and central regions. Farms with an average of ≥24 pigs weaned per sow per year exhibited a higher prevalence of *C. difficile* than those weaning fewer than 24 pigs/sow/year (p < 0.05). Furthermore, farms administering mixed antibiotics via feed during the peripartum period showed significantly higher risk for *E. coli* presence (p = 0.01).

**Table 3:** Potential risk factors for pathogens identified in the survey (statistically significant p *≤* 0.05).

Risk factor	Pathogen	% High positive (n)	% Low positive (n)	% Negative (n)	p-value
Average number of weaned pigs					
≤24	*C. difficile*	16.13 (5)	41.94 (13)	41.94 (13)	≤0.05
>24		62.50 (5)	12.50 (1)	25 (2)	
Peripartum antibiotic administration in sows					
Injected	*E. coli*	5 (1)	50 (10)	45 (9)	≤0.01
Mix feed		26.32 (5)	57.89 (11)	15.79 (3)	
Farm type					
Farrowing sow	*C. perfringens*	41.67 (5)	58.33 (7)	0	≤0.05
2@Farrow-to-finish		75 (21)	25 (7)	0	
Geographical region					
North	*C. difficile*	43.75 (7)	18.75 (3)	37.5 (6)	≤0.01
Central		50 (3)	16.67 (1)	33.33 (2)	
South		0	55.56 (10)	44.44 (8)	

*E. coli=Escherichia coli, C. perfringens=Clostridium perfringens, C. difficile=Clostridioides difficile*

Trends suggesting potential associations were observed between *C. difficile* prevalence and certain management practices, such as a higher number of piglets born alive, split suckling, and younger parities (Table 4). Other investigated variables were not found to significantly influence pathogen prevalence.

**Table 4:** Potential risk factors for pathogens in the survey showed trends (p ≤ 0.10).

Risk factor	Pathogen	% High positive (n)	% Low positive (n)	% Negative (n)	p-value
Number of piglets born alive					
≤25	*C. difficile*	14.29 (3)	38.10 (8)	47.62 (10)	≤0.10
>25		38.89 (7)	33.33 (6)	27.78 (5)	
Split suckling practices					
Yes	*C. perfringens*	85.71 (12)	14.29 (2)	0	≤0.10
No		53.85 (14)	46.15 (12)	0	
Yes	*C. difficile*	50 (7)	14.29 (2)	35.71 (5)	≤0.10
No		11.54 (3)	46.15 (12)	42.31 (11)	
Younger parity are more affected					
Yes	*C. difficile*	40.91 (9)	27.27 (6)	31.82 (7)	≤0.10
No		5.88 (1)	47.06 (8)	47.06 (8)	

*C. perfringens=Clostridium perfringens, C. difficile=Clostridioides difficile*

#### Relationship between number of pathogens and farm variables

Several farm variables were significantly associated with a higher number of pathogen types, including region, farm size, weaning weight, and the percentage of piglets moved after 2 days of age (Table 5). Farms located in northern Vietnam had a higher proportion of samples positive for two or three pathogens compared to farms in the central and southern regions (p = 0.05). In addition, farms with more than 1,000 sows exhibited a higher frequency of samples containing multiple pathogens (p = 0.05). A greater number of pathogens was also linked to lower average weaning weights (p = 0.05). Finally, farms moving ≥10% of piglets by the second day of lactation showed a higher prevalence of multiple pathogens.

**Table 5:** Potential risk factors associated with multiple pathogens (statistically significant p *<* 0.05).

Risk factor	Total number of pathogens			p-value

	1 pathogen (%)	2 pathogens (%)	3 pathogens (%)	
Geographical region				
North	21.28	55.32	23.4	≤0.05
Central	42.86	28.57	28.57	
South	45.28	41.51	13.21	
Size				
≤1000	42.42	40.91	16.67	≤0.05
>1000	25	52.08	22.92	
Weaning weight				
<6	15.79	52.63	31.58	≤0.05
6–6.5	30.30	45.45	24.24	
>6.5	50	34.38	15.62	
Piglets moved after 2 days				
≤10%	43.06	40.28	16.67	≤0.05
>10%	16.67	66.67	16.67	

A trend (0.05 < p ≤ 0.10) was observed between “type of farm (farrow-to-finish)” and “antibiotic route (mixed feed)” and an increased number of pathogen types (Table 6). Other variables assessed in the survey did not show significant associations with pathogen prevalence.

**Table 6:** Potential risk factors associated with multiple pathogens were identified in the survey showed trends (p < 0.10).

Risk factor	Total number of pathogens			p-value

	1 pathogen (%)	2 pathogens (%)	3 pathogens (%)	
Type of farm			
Farrowing sow	57.58	27.27	15.15	≤0.10
Farrowing- finishing	25.93	53.09	20.99	
Antibiotic route				
Injected	42.59	42.59	14.81	≤0.10
Mix feed	29.31	46.55	24.14	

## DISCUSSION

Neonatal diarrhea remains a significant health challenge within swine production systems, with multiple pathogens identified as causative agents, including *C. perfringens*, *E. coli*, and *C. difficile* [25]. The findings of our study confirmed that these pathogens are highly prevalent and present a substantial risk to pig farms in Vietnam. Notably, *C. perfringens* type A was universally detected at the farm level (100%) and was present in 95.61% of the total samples. This prevalence surpasses rates reported in previous studies by Vidal *et al*. [9] and Mesonero-Escuredo *et al*. [26], which documented infection rates ranging from 70.7% to 89.9%. Moreover, *C. perfringens* types C and D were found to co-occur with type A in 2.5% of farms. These observations align with previous reports from Thailand, where similarly high prevalence levels were recorded [27].

The detection of *C. difficile* as a potential emerging pathogen represents a particularly novel and important finding, underscoring the evolving complexity of pathogen interactions in neonatal piglet diarrhea. Although *C. difficile* has already been identified as an emerging pathogen in neonatal diarrhea in other regions [28–30], its role in piglet diarrhea within the Vietnamese context has not been extensively studied. The present study offers compelling evidence that *C. difficile* may be more prevalent than previously recognized, particularly in the case of suckling piglets. This raises pertinent questions about its pathogenic role and its potential contribution to the severity and recurrence of diarrhea in Vietnamese piglets.

The elevated detection of *C. difficile* in diarrheic piglets – identified on 60% of infected farms – indicates its potential as a notable contributor to the clinical manifestation of diarrheal disease. This is especially relevant for farms experiencing recurrent or severe episodes, where *C. difficile* may exacerbate clinical symptoms [31]. Neonatal diarrhea is a multifactorial disease frequently involving multiple pathogens, and the presence of *C. difficile* in this study further complicates diagnostic and management considerations. Its detection in 92.5% of farms as part of co-infection scenarios, frequently in association with *C. perfringens* type A, suggests that *C. difficile* may not act independently but could interact with other microbial agents to intensify the diarrheal disease process [32]. This finding challenges the previously held assumption that neonatal diarrhea is predominantly caused by single-pathogen infections. Our study demonstrated that polymicrobial infections are often involved in neonatal diarrhea outbreaks, with three-pathogen combinations comprising 35% of cases, compared to only 10% involving a single pathogen.

A particularly significant and novel observation was the high frequency of coinfections involving multiple toxin-producing bacterial agents. The consistent presence of *C. perfringens* type A in all co-infected samples suggests potential synergistic interactions between this pathogen and others. As a well-documented enteric pathogen in swine, the concurrent detection of *C. perfringens* type A and *C. difficile* may potentiate the virulence and disease severity. Furthermore, the predominance of three-pathogen combinations (35%) reinforces the hypothesis that neonatal diarrhea in piglets is not typically the result of a singular infectious agent, but rather a complex interplay of diverse pathogens [33].

Logistic regression analysis from this study highlights several key risk factors influencing the presence of neonatal diarrhea pathogens at the herd level. Farm characteristics such as operation type, geographic location, antibiotic use practices, and piglet weaning metrics were significantly correlated with elevated prevalence of *C. perfringens*, *E. coli*, and *C. difficile*. For instance, farrow-to-finish farms – which rear pigs from birth through to market weight – were more likely to exhibit higher pathogen loads, potentially due to the continuous animal turnover and associated risk of pathogen persistence and transmission [34]. In addition, geographical differences in *C. difficile* prevalence suggest that regional factors such as climate or specific management practices may influence pathogen ecology. In particular, *C. difficile* was less frequently detected in farms located in southern Vietnam, which could reflect differing biosecurity strategies or environmental conditions affecting bacterial survival.

The study also underscores the contrast between open-house and closed-house production systems in Vietnam. Closed-house systems typically incorporate environmental controls and technology-driven management practices that minimize exposure to pathogens. Conversely, open-house farms, which lack such infrastructure, are more vulnerable to disease outbreaks due to fluctuating environmental factors and increased risk of external pathogen introduction [35].

Collectively, the findings reinforce the necessity of adopting a comprehensive and multifaceted approach to mitigate neonatal diarrhea and its impacts. A cornerstone of this strategy includes the development and implementation of effective vaccination programs for sows [36]. Immunization facilitates the passive transfer of antibodies to piglets through colostrum, offering protection during the critical early postnatal period [8, 26]. Beyond vaccination, the adoption of innovative management practices – such as improved environmental regulation and rigorous hygiene protocols – is also essential for effective disease control [36, 37]. Ensuring proper housing, sanitation, and biosecurity can significantly reduce the introduction and spread of enteric pathogens, thereby supporting improved piglet health and production outcomes.

## CONCLUSION

This study presents the first large-scale epidemiological assessment of toxin-producing bacterial pathogens associated with neonatal diarrhea in piglets across commercial sow farms in Vietnam. The findings reveal a high prevalence of *C. perfringens* type A (95.61%), *E. coli* (46.49%), and *C. difficile* (39.47%) among diarrheic piglets, with *C. perfringens* detected in all co-infected cases and present on every farm surveyed. Notably, *C. difficile* emerged as a potentially underrecognized pathogen, identified in 60% of infected farms and frequently associated with co-infection events. Moreover, the detection of multiple virulence genes – such as LT toxin (*E. coli*), alpha toxin (*C. perfringens*), and A-toxin (*C. difficile*) – highlights the pathogenic complexity and reinforces the multifactorial nature of neonatal diarrhea.

The strength of this study lies in its comprehensive design, which integrates molecular diagnostics through multiplex qPCR with structured farm-level surveys to elucidate risk factors associated with pathogen occurrence. Significant associations were identified between pathogen prevalence and farm type, geographic region, peripartum antibiotic administration, piglet weaning performance, and housing system. The large sample size, representative geographic coverage, and identification of co-infection dynamics add substantial value to veterinary epidemiology in Southeast Asia.

However, several limitations warrant consideration. The cross-sectional nature of the study limits causal inference between identified risk factors and pathogen prevalence. In addition, the use of convenience sampling may introduce selection bias, potentially affecting the generalizability of results to the wider swine population. Furthermore, the absence of viral and protozoal pathogen analysis restricts a full understanding of the etiological spectrum of neonatal diarrhea.

Future studies should adopt longitudinal designs with randomized sampling to establish causal relationships and monitor pathogen dynamics over time. Investigations incorporating viral, parasitic, and environmental components will provide a more holistic understanding of neonatal diarrhea epidemiology. Moreover, intervention-based studies evaluating the efficacy of targeted vaccination, optimized antibiotic stewardship, and enhanced biosecurity protocols are essential for developing evidence-based control strategies.

This study offers critical insights into the bacterial ecology of neonatal diarrhea in Vietnamese pig production and underscores the need for integrated, region-specific prevention, and management approaches to safeguard piglet health and improve farm productivity.

## DATA AVAILABILITY

The supplementary data (Table S1 and S2) can be available from the corresponding author on a reasonable request.

## AUTHORS’ CONTRIBUTIONS

DTD and JME: Designed the study. NMD, DTD, HDV, and TTNN: Performed experiments. JME, ST, DCL, and TTNN: Analyzed the data. DTD, JME, TTNN, and DCL: Wrote the manuscript. All authors have read and approved the final manuscript.
